# Exploring Experimental and In Silico Approaches for Antibody–Drug Conjugates in Oncology Therapies

**DOI:** 10.3390/ph18081198

**Published:** 2025-08-14

**Authors:** Vitor Martins de Almeida, Milena Botelho Pereira Soares, Osvaldo Andrade Santos-Filho

**Affiliations:** 1 Laboratory of Molecular Modeling and Computational Structural Biology, Walter Mors Natural Products Research Institute, Health Science Center, Federal University of Rio de Janeiro, Av. Carlos Chagas Filho, 373, Bloco H, Cidade Universitária, Rio de Janeiro 21941-599, RJ, Brazil; vitoralmeida1808@ufrj.br; 2 Gonçalo Moniz Institute, Oswaldo Cruz Foundation, Rua Waldemar Falcão, 121, Salvador 40296-710, BA, Brazil; milena.soares@fiocruz.br; 3 Institute of Innovation in Advanced Health Systems (ISI SAS), University SENAI CIMATEC, Av. Orlando Gomes, 1845, Salvador 41650-010, BA, Brazil

**Keywords:** antibody–drug conjugates, cancer, linker, payload, molecular modeling, ADC design, targeted therapy

## Abstract

Background/Objectives: Antibody–drug conjugates are a rapidly evolving class of cancer therapeutics that combine the specificity of monoclonal antibodies with the potency of cytotoxic drugs. This review explores experimental and computational advances in ADC design, focusing on structural elements and optimization strategies. Methods: We examined recent developments in the mechanisms of action, antibody engineering, linker chemistries, and payload selection. Emphasis was placed on experimental strategies and computational tools, including molecular modeling and AI-driven structure prediction. Results: ADCs function through both internalization-dependent and -independent mechanisms, enabling targeted drug delivery and bystander effects. The therapeutic efficacy of ADCs depends on key factors: antigen specificity, linker stability, and payload potency. Linkers are categorized as cleavable or non-cleavable, each with distinct advantages. Payloads—mainly tubulin inhibitors and DNA-damaging agents—require extreme potency to be effective. Computational methods have become essential for antibody modeling, developability assessment, and in silico optimization of ADC components, accelerating candidate selection and reducing experimental labor. Conclusions: The integration of experimental and in silico approaches enhances ADC design by improving selectivity, stability, and efficacy. These strategies are critical for advancing next-generation ADCs with broader applicability and improved therapeutic indices.

## 1. Introduction

Cancer remains a significant global public health challenge and is the second leading cause of death worldwide. The International Agency for Research on Cancer (IARC) estimated approximately 20 million new cancer cases and 9.7 million cancer-related deaths globally in 2022, suggesting that about one in five individuals will develop cancer during their lifetime, with approximately one in nine men and one in twelve women succumbing to the disease [1]. Despite advancements in therapeutic options, conventional treatments such as chemotherapy and radiotherapy continue to face significant limitations that compromise their efficacy and increase the risk of adverse effects. One primary challenge of these approaches is their lack of specificity, leading to the destruction of healthy cells alongside malignant cancer cells. This indiscriminate effect results in systemic damage to normal tissues, contributing to toxicity and debilitating side effects, including immunosuppression, fatigue, and gastrointestinal issues [2]. Furthermore, many tumors develop resistance to conventional treatments over time, rendering them ineffective in advanced stages of the disease. Another significant issue is the insufficient penetration of chemotherapeutic agents in certain types of tumors, particularly solid tumors with complex microenvironments, limiting the treatment’s ability to effectively target malignant cancer cells in deeper tissue regions [2,3]. These challenges show the urgent need for more targeted and effective cancer therapies that can overcome the limitations of current treatment modalities.

**Figure 1:**
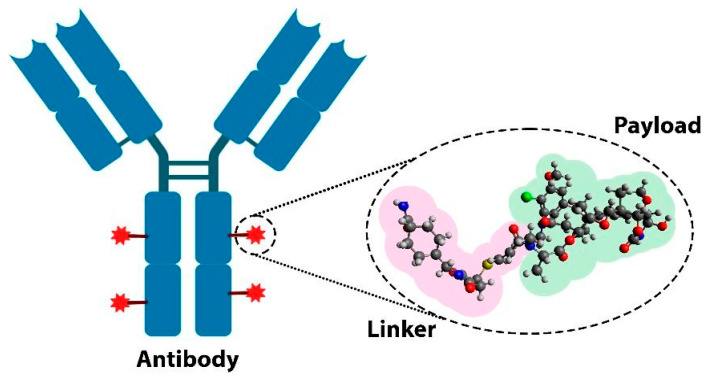
Schematic representation of the components of an antibody–drug conjugate: a monoclonal antibody, a cytotoxic drug, and a molecular linker.

Antibody–drug conjugates (ADCs) represent a groundbreaking advancement in cancer therapy, combining the specificity of monoclonal antibodies with the potent cytotoxic effects of chemotherapy. ADCs consist of three essential components: a monoclonal antibody (mAb), which has the ability to selectively bind to specific antigens (e.g., proteins) expressed on cancerous cells; a payload (or cytotoxic drug), capable of inducing tumor cell death; and a molecular linker that connects the antibody to the drug (Figure 1). This three-piece structure allows ADCs to deliver the drug directly to tumor cells, minimizing damage to surrounding healthy tissues and thus reducing the systemic side effects commonly associated with traditional chemotherapy treatments [4,5].

The importance of ADCs in the treatment of cancer patients lies in the ability of the antibody to selectively bind to specific antigens on cancerous cells. By binding to antigens that are overexpressed on cancer cells but absent or minimally present on healthy cells, ADCs enhance therapeutic efficacy while sparing healthy tissues [4,5,6]. This targeted approach not only improves the overall effectiveness of cancer treatments but also offers a more favorable safety profile compared to conventional therapies. From May 2000 to June 2023, 13 ADCs had been approved by regulatory agencies in the United States, European Union, and China for various cancer types, including breast, lung, and ovarian cancers, demonstrating their clinical utility across a range of malignancies [7].

Advancements in the development of ADCs have clearly demonstrated their potential to overcome the limitations of conventional cancer therapies by providing more targeted treatments with reduced side effects. In this review, we explore the multifaceted mechanisms of ADCs—from antigen recognition and binding to the controlled intracellular release of the cytotoxic payload—while evaluating current challenges such as optimizing linkers, selecting appropriate payloads, and overcoming therapeutic resistance. Moreover, we examine the emerging role of computational methods and AI-driven platforms in ADC research. These innovative approaches are being used to predict antibody–antigen interactions, optimize linker stability, and forecast payload performance, ultimately reducing development timelines while enhancing therapeutic precision through personalized medicine. This integrated discussion paves the way for an in-depth look at the latest advances in bioengineering, in silico techniques, and natural product libraries that are shaping the future of ADC therapy.

## 2. Mechanism of Action of ADCs in Cancer

**Figure 2:**
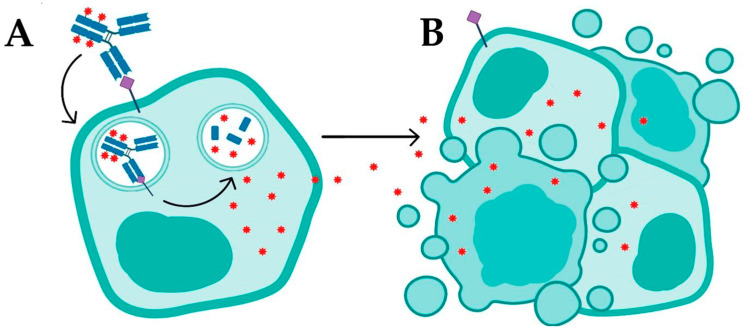
Mechanisms of action of antibody–drug conjugates: (**A**) targeted drug delivery via antigen–antibody recognition and internalization, leading to apoptosis or necrosis; and (**B**) bystander killing of adjacent tumor cells through passive diffusion of lipophilic drugs.

ADCs operate based on complex mechanisms of action, which can be divided into two general processes: internalization and non-internalization of the ADC (Figure 2). Internalization is the primary mechanism for ADC function, where the process begins with the binding of the monoclonal antibody to a specific antigen on the surface of target tumor cells (Figure 2A). This specific antigen–antibody recognition is crucial for directing the ADC to its intended target and initiates receptor-mediated endocytosis. In many cases, this endocytic process involves various pathways, such as clathrin-mediated or caveolin-mediated endocytosis, which facilitate the transport of the ADC complex into the cell. After binding, the antigen–ADC complex is internalized by the cell via receptor-mediated endocytosis (Figure 2A). During this process, the complex is transported through intracellular compartments, such as endosomes, and ultimately to lysosomes, where the acidic environment facilitates the enzymatic or chemical cleavage of the linker connecting the antibody to the cytotoxic drug (Figure 2A).

The design and chemical properties of the linker are essential at this stage; it must be stable during systemic circulation yet readily cleavable in the intracellular environment to ensure timely release of the payload. In the lysosome, the cleavage of the linker releases the cytotoxic drug, which diffuses into the cytoplasm and interferes with critical cellular components—such as DNA or microtubules—leading to cell death via apoptosis or necrosis (Figure 2B) [4,5,8]. This internalization process is particularly effective in tumors with high antigen expression, enabling the direct delivery of the drug into target cells; however, its efficacy may be limited in tumors with heterogeneous antigen expression or in cells that do not internalize ADCs efficiently [5,9].

In addition to internalization, ADCs can operate via a non-internalization mechanism that serves as an alternative and complementary pathway. This mechanism involves the release of the cytotoxic payload into the tumor microenvironment, allowing the drug to act on adjacent tumor cells. In this context, neighboring cells may not express the target antigen (Ag^−^), or even if they express the antigen (Ag^+^), they may fail to internalize the ADC complex [10]. This phenomenon, known as “adjacent cell death”, is primarily observed with ADCs that incorporate lipophilic drugs, which can readily cross cellular membranes and diffuse into nearby cells. In tumors with heterogeneous antigen expression, this property can enhance therapeutic efficacy by enabling the drug to target a larger number of malignant cells independent of direct internalization [10]. ADCs with cleavable linkers are particularly effective in this context, as they can release the drug into the extracellular milieu via distinct mechanisms—such as the reduction of disulfide bonds (-S-S-), pH-driven chemical or enzymatic hydrolysis, or proteolytic cleavage—thus promoting payload release without the necessity for internalization [10,11]. Recent advances in ADC technology have further expanded this strategy by focusing on targeting various components of the tumor microenvironment, including the extracellular matrix, stromal cells, and neovasculature. This approach is particularly promising for solid tumors or those exhibiting low internalization rates, thereby broadening the therapeutic applicability of ADCs to tumor types that were previously challenging to treat with traditional ADC mechanisms [12,13].

### Antibody Selection in Extracellular Matrix (ECM)-Targeted ADCs

Extracellular matrix ECM-targeted antibody–drug conjugates are an emerging class of therapeutic agents designed to selectively deliver cytotoxic agents to the tumor ECM rather than relying on cellular internalization. These ADCs target specific antigens abundantly expressed in the tumor ECM, such as splice isoforms of tenascin-C, enabling precise localization to the subendothelial matrix within tumors. Upon binding, the conjugated cytotoxic payload is released extracellularly through proteolytic cleavage of linkers, such as valine–citrulline dipeptide linkers, by proteases present in the tumor microenvironment. The extracellular drug release induces potent anti-cancer effects, leveraging the bystander killing of nearby tumor cells without requiring antibody internalization. This strategy expands the ADC therapeutic scope to antigens not readily internalized, offering novel opportunities to target the tumor microenvironment and improve treatment efficacy with reduced impact on normal tissues [14,15]. Since antibodies can localize selectively to specific tissues, ECM-targeted antibody–drug conjugates offer a strategic advantage to facilitate delivery of cytotoxic agents while minimizing off-target toxicity. This concept has been well demonstrated in oncology, where ADCs such as trastuzumab emtansine (T-DM1) combine targeted antibodies with potent payloads like emtansine, enabling tissue-specific drug retention and enhanced therapeutic efficacy that were not achievable with untargeted agents due to systemic toxicity [16]. Translating this approach beyond cancer, ADCs are now being explored for inflammatory diseases such as arthritis, where selective delivery of therapeutic agents to diseased tissues, including the extracellular matrix of joints, could improve outcomes [17]. Moreover, fusion proteins known as immunocytokines, which couple cytokines to antibodies that home to disease sites, have shown potential to increase local cytokine concentrations while reducing systemic side effects. For instance, antibody–cytokine conjugates using pro-inflammatory IL-2 have shown promise in oncology, and similarly, ADCs carrying anti-inflammatory cytokines such as IL-4 and IL-10 are in development for rheumatoid arthritis [17,18]. Collectively, these advances illustrate how antibody-based targeting strategies that exploit tissue- and ECM-specific antigens can improve delivery of both cytotoxic and immunomodulatory payloads, broadening the therapeutic applications and enhancing the safety profile of ADCs.

Effective design of ECM-targeted ADCs requires careful consideration of antibody properties that govern payload distribution and therapeutic efficacy within the tumor microenvironment. Recent peer-reviewed studies and mechanistic models have provided crucial insights into how key antibody properties—namely tumor penetration, internalization rate, affinity versus retention, ECM antigen heterogeneity, and Fc receptor interactions—influence ADC pharmacokinetics, payload delivery, and clinical efficacy [19,20].

A fundamental consideration in the development of ECM-targeting ADCs involves optimizing tumor penetration, which is strongly influenced by antibody affinity. Although high-affinity antibodies are traditionally favored for their strong target binding, they may paradoxically hinder deep tumor penetration due to the “binding site barrier” effect. In this scenario, antibodies become trapped near tumor vasculature, preventing efficient diffusion into more distal tumor regions. Moderate- or even low-affinity antibodies have demonstrated superior tissue distribution in dense and heterogeneous ECM environments, maximizing payload exposure across the tumor mass. Mechanistic PK/PD models support this notion, indicating that tuning antibody affinity to intermediate levels enhances delivery efficacy, especially in solid tumors where ECM antigen expression is spatially variable [21].

The internalization rate of ADC–antigen complexes, a key factor in intracellular payload delivery, plays a markedly different role when the target resides in the extracellular matrix (ECM). Unlike internalizing cell surface antigens, most ECM components do not internalize, requiring ADCs designed for extracellular payload release and deep tissue penetration. Classical ADCs that favor rapid internalization may limit tumor distribution due to perivascular binding. Nessler et al. showed that, in a colorectal carcinoma model, using an anti-CEA ADC (SN-38 conjugate) that enhanced internalization improved efficacy only at clinically saturating doses. Co-administration of a cross-linking anti-CEA antibody reduced the internalization half-life (~14.5 h to ~5 h), improving intracellular delivery and tumor killing. While some bystander effect was noted, intracellular release was the primary driver of efficacy under these conditions [22]. In contrast, for non-internalizing ECM targets, reduced internalization favors wider diffusion before extracellular payload release. In these cases, ADCs must use membrane-permeable payloads with strong bystander activity, and linker chemistry should support extracellular activation rather than lysosomal release [22].

Adding further complexity, the spatial heterogeneity of ECM antigen expression alters both ADC binding dynamics and therapeutic distribution. In low-antigen regions, ADCs may not accumulate sufficiently unless their payloads exhibit bystander killing. Conversely, high antigen expression near vasculature can lead to perivascular sequestration and limited tissue penetration. For example, Khera et al. used cellular-resolution pharmacodynamic mapping to study an anti-GCC ADC (TAK-164) and its lipophilic DNA-alkylating payload DGN549 in 3D tumor spheroids and primary xenografts. They showed that the lipophilicity of DGN549 enables penetration beyond antigen-positive cell layers into adjacent antigen-negative regions—a key demonstration that diffusible payloads can overcome spatial heterogeneity in antigen expression [23].

Additionally, Ascione et al. emphasize that antigen expression measured from a single biopsy may not represent the full heterogeneity across tumor sites. They note that inconsistent antigen levels across regions complicate ADC efficacy prediction and patient selection—and underscore the need for payload properties that can compensate for such heterogeneity [24]. These studies consistently show that effective ECM-targeting ADCs often pair antibodies with diffusible payloads capable of penetrating into low-antigen stromal zones and inducing cytotoxicity beyond immediate binding sites.

Although Fc-mediated effector functions are traditionally leveraged in ADCs targeting cell surface markers, their role in ECM-targeting ADCs is more nuanced. Since ECM antigens reside in the cell-free matrix, direct antibody-dependent cell-mediated cytotoxicity (ADCC) may play a limited role [17]. However, Fc engineering remains relevant due to its impact on antibody half-life, systemic clearance, and tissue retention. Modifications such as afucosylation, isotype switching, and glycosylation tuning can modulate Fcγ receptor engagement and indirectly influence payload exposure by altering biodistribution kinetics. Therefore, while immune recruitment may be secondary, Fc design continues to influence the pharmacokinetic profile of ECM-targeting ADCs [17,25].

Integrating computational modeling with empirical data has proven invaluable for refining ADC design strategies. Predictive models that incorporate variables such as antigen density, antibody affinity, internalization rate, and payload diffusivity have demonstrated strong concordance with in vivo outcomes, surpassing traditional metrics such as plasma concentration alone [26,27,28]. Xenograft studies confirm that payload distribution within the tumor interstitium—not merely total antibody accumulation—correlates most strongly with antitumor efficacy [29]. These findings underscore the importance of tailoring antibody properties not only for antigen recognition but also for their interaction with the dynamic tumor microenvironment, enabling rational, model-informed design of ECM-targeting ADCs with improved therapeutic indexes.

## 3. Monoclonal Antibodies in ADCs

The ability of ADCs to effectively target the tumor microenvironment and adjacent cells is largely attributed to the choice of the monoclonal antibody employed. The antibody not only directs the cytotoxic payload to target cells but also modulates the immune response and influences overall treatment efficacy. Its specificity for tumor antigens is critical, as it determines the selectivity of the ADC and minimizes toxicity to healthy tissues [30]. Moreover, the interaction between the antibody and the tumor microenvironment can affect internalization dynamics and promote desirable bystander effects, such as adjacent cell death [31]. Thus, a detailed examination of the monoclonal antibody is essential for understanding how ADC-based therapies can be optimized to offer more effective and safer treatments for patients, particularly in tumors with challenging biological characteristics.

**Figure 3:**
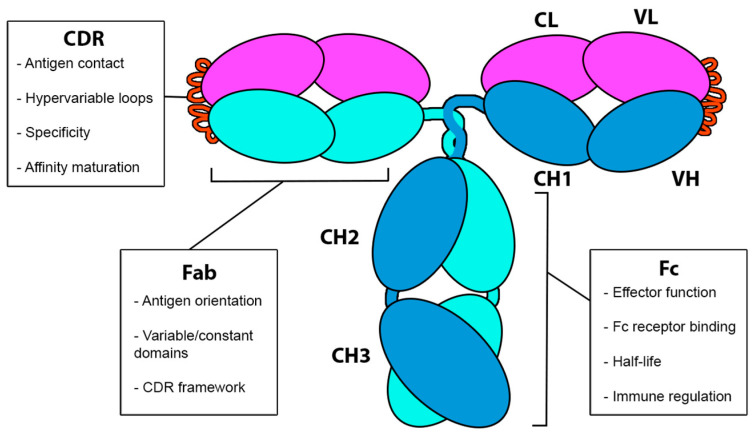
General structure of an antibody, which consist of two light chains (L, light, in magenta) and two heavy chains (H, heavy, in cyan and blue). The Fab region comprises the variable domains VH (variable, heavy) and VL (variable, light), along with the constant domains CH1 (constant, heavy) and CL (constant, light). The Fc region consists of the constant domains CH2 and CH3. CDR loops are highlighted in orange.

As shown in Figure 3, human antibodies, such as immunoglobulin G (IgG), are glycoproteins composed of two identical light chains (LCs) and two identical heavy chains (HCs) that associate to form a Y-shaped monomer. The light chains consist of a constant domain (CL, light chain constant domain) and a variable domain (VL, light chain variable domain). Similarly, the heavy chains are composed of a constant domain (CH, heavy chain constant domain) and a variable domain (VH, heavy chain variable domain). The structure of the antibody can be divided into two main regions: the antigen-binding fragment (Fab), responsible for antigen recognition, and the crystallizable fragment (Fc), which interacts with immune cell receptors and mediators of the immune system. Within the Fab region, the complementarity-determining regions (CDRs) are hypervariable loops that confer specificity to the antibody, enabling it to recognize a wide array of antigens. Understanding the relationship between antibody structure and function is fundamental for achieving the desired therapeutic response, especially considering the importance of affinity and avidity in the efficacy of interactions between the antibody and target antigens [32,33].

The CDRs of the heavy and light chains can be classified as CDR1, CDR2, and CDR3, forming the primary antigen-binding site and exhibiting significant structural diversity, particularly in the CDR3 segment of the heavy chain, which is typically longer and more variable. Chemically, CDRs are characterized by the presence of amino acid residues whose composition and spatial distribution are critical in establishing hydrophobic, ionic, and hydrogen bond interactions with tumor antigen epitopes. The structural arrangement of CDRs enables a unique combination of non-covalent interactions with the antigen, where the binding affinity is largely dictated by the complementarity between interacting surfaces [33,34]. In practice, the side chains of residues within the CDRs can reorient in response to the epitope, allowing for a fine-tuned adjustment known as induced fit. The CDR3 of the heavy chain is particularly prone to structural adaptation, providing enhanced flexibility to accommodate antigens of varying sizes and serving as the most critical region for affinity optimization in antibodies [33,34,35]. Molecular dynamics simulations have demonstrated that intermolecular interactions are influenced by the pH of the tumor microenvironment, which affects the protonation state of titratable residues and, consequently, the spatial configuration of CDRs and their binding capacity [36]. Crystallographic analyses have revealed that CDRs form binding “hotspots,” consisting of residues that contribute disproportionately to binding affinity. These regions are of particular interest in antibody engineering for ADCs, as they allow for selective modification of residues to enhance antigen binding without compromising the structural stability of the molecule [37,38].

Despite the high specificity and strong antigen-binding affinity of antibodies that make them ideal for ADCs, their efficacy against solid, densely packed tumors remains constrained. The large molecular weight of IgG (~150 kDa) significantly limits tumor microenvironment penetration, particularly in malignancies with a compact extracellular matrix. Beyond its sheer size, the slow diffusion of IgG through physical barriers—such as collagen networks and proteoglycan-rich stroma—further restricts tissue distribution [4,19]. These structural constraints are compounded by the elevated interstitial fluid pressure commonly observed in solid tumors, which reduces passive transport of macromolecules. Consequently, IgG-based ADCs often fail to deliver sufficient cytotoxic payload to cells in the tumor core, undermining their therapeutic efficacy [39,40].

To mitigate the challenges associated with the large size of antibodies, recent studies have increasingly explored smaller monoclonal antibody formats, such as antigen-binding fragments (Fabs), single-chain variable fragments (scFvs), and nanobodies [41,42]. Due to their lower molecular weight and compact structure (~15 kDa for nanobodies), these alternatives exhibit markedly improved tumor penetration compared to full-length IgG antibodies (~150 kDa). For example, Debie et al. demonstrated in vivo, using intravital fluorescence microscopy, that monomeric nanobodies rapidly extravasate and distribute homogenously throughout HER2-positive tumor tissue within minutes post-injection, achieving maximal tumor uptake significantly faster than trastuzumab, which exhibited slower, perivascular, and heterogeneous distribution even after 24 h. This rapid and homogeneous penetration is primarily attributed to the small size and monovalent binding of nanobodies, which overcome the “binding site barrier” effect commonly observed with full-length antibodies due to their high avidity and larger size [43]. Importantly, this enhanced intratumoral dissemination has been correlated with improved therapeutic outcomes in preclinical models, as more uniform tumor coverage can reduce the likelihood of untreated cancer cell populations, especially relevant for therapies with short diffusion ranges such as antibody–drug conjugates [43,44,45].

Additionally, the smaller formats typically exhibit shorter serum half-lives due to the absence of the Fc region and consequent lack of FcRn-mediated recycling, resulting in faster systemic clearance. This short half-life can be partially addressed through fusion to serum albumin or polyethylene glycol (PEG), although such modifications increase molecular size and may reduce the penetration advantages of smaller formats while raising production costs. Beyond structural optimization, in silico and experimental antibody engineering continues to focus on enhancing specificity, affinity, and stability to maximize clinical utility [42,44].

A second example of enhanced penetration by smaller antibody formats is demonstrated with a cell-penetrating single-chain variable fragment (CPP-scFv) engineered to target mutant HRas (G12V). This hyperstable scFv format, approximately 28 kDa in size, was fused to a cell-penetrating peptide (CPP), enabling rapid internalization and intracellular engagement of Ras—a target inaccessible to full-length IgG antibodies due to their bulk and lack of membrane permeability. In vivo binding assays confirmed that CPP-scFv(Ras) successfully crossed the cell membrane and localized to its intracellular antigen, validating functional penetration in a reducing environment. Compared to conventional antibodies restricted to extracellular targets, this scFv-based design illustrates how a reduced molecular weight combined with CPP-mediated delivery can overcome penetration barriers and enable effective intracellular targeting of oncogenic proteins [46].

However, it is important to acknowledge that, while these preclinical data provide compelling evidence of superior tumor penetration by smaller antibody formats, comprehensive pharmacokinetic and pharmacodynamic studies establishing a direct quantitative link between improved penetration and clinical efficacy remain limited. Further in vivo investigations and clinical trials are required to confirm how these differences translate into therapeutic benefit.

To enhance the efficacy of monoclonal antibodies, advanced molecular biology techniques such as phage display have proven exceptionally powerful. This method leverages bacteriophages to present diverse antibody fragments on their surface. By exposing a highly variable phage library to a specific antigen, it enables the selective enrichment of phages displaying high-affinity antibody variants through iterative rounds of binding and amplification [47,48]. The result is a remarkably precise and adaptable approach that facilitates the identification of antibodies targeting a wide spectrum of antigens, including complex tumor-associated molecules, thereby accelerating the development of highly specific therapeutic candidates. In parallel, related display technologies—such as ribosome and yeast display—have gained traction in antibody screening and engineering, offering complementary strategies to increase efficiency in the preclinical development pipeline [48,49]. However, despite their strengths, these platforms also present notable limitations: they rely on biological systems that may not fully capture the intricacies of in vivo antibody–antigen interactions, and the screening procedures can be time-consuming and labor-intensive, often requiring extensive optimization. These challenges are compounded by difficulties in isolating antibodies with optimal pharmacological properties and by technical issues such as aggregation or instability of antibody fragments during display processes [48,50].

Concurrently with advances in experimental strategies, the optimization of antibody structures through in silico molecular modeling techniques has made significant strides. Computational methods have gained prominence in recent years, playing an essential role in predicting and refining antibody–antigen interactions, with the goal of enhancing binding specificity and affinity. Some of these techniques have become the cornerstone of many antibody development pipelines.

### Antibody Structure Modeling

Recent advances in antibody structural modeling have been driven by the development of deep learning frameworks capable of predicting three-dimensional conformations directly from amino acid sequences, a capability that becomes indispensable when no experimental (X-ray, NMR, and Cryo-EM) structure is available. Initially, homology-based methods such as RosettaAntibody exploited curated libraries of Fv domain structures to graft framework and CDR loop templates, accurately recapitulating canonical loop conformations and yielding predicted CDR-loop RMSDs of <1.5 Å—a performance that enabled reliable modeling in the absence of crystal templates [51]. In parallel, software like ABodyBuilder3 represents a significant advancement in antibody structure prediction, building upon its predecessor, ABodyBuilder2. It achieves state-of-the-art accuracy in modeling complementarity-determining region (CDR) loops by leveraging language model embeddings. The model incorporates eight sequential and independent update blocks that process an embedding representation of the variable region sequence to predict final atomic coordinates and uncertainties. To enhance the quality of predicted structures, ABodyBuilder3 employs relaxation strategies using tools like OpenMM [52] or YASARA2 [53]. Additionally, it integrates a predicted Local Distance Difference Test (pLDDT) into its output, allowing for more accurate estimation of uncertainties. These enhancements collectively enable scalable, template-free modeling of diverse antibody repertoires directly from sequence data [54].

The introduction of generalist predictors marked a turning point in antibody modeling by removing the dependency on closely related templates. AlphaFold 2 (AF2) leveraged multiple-sequence alignments, attention-based encoders, and end-to-end coordinate optimization to achieve atomic-level accuracy, with a median backbone RMSD of approximately 0.96 Å on CASP14 benchmarks [55]. This advancement enabled de novo modeling of novel antibody sequences without available homologous structures. Although AF2 exhibited exceptional accuracy across diverse proteins, its initial benchmarking on antibody–antigen complexes revealed challenges in modeling hypervariable CDR loops and interface conformations without specialized training data [56]. To address these limitations, antibody-focused extensions emerged: xTrimoABFold replaces MSA-dependent evoformer blocks with a transformer-based antibody language model, achieving over 30% RMSD improvement on CDRs and predictions 150 times faster than AF2—critical advantages when rapid, template-free modeling is needed [57]. IgFold employs embeddings from a 558 million-sequence AntiBERTy language model to predict Fv coordinates end-to-end in under 25 s, matching AF2 accuracy while providing per-residue confidence scores that guide refinement without experimental structures [58].

Subsequent tools have further broadened the computational repertoire for template-free antibody design. ABlooper exemplifies this progression by employing E(n)-Equivariant Graph Neural Networks (E(n)-EGNNs) to predict CDR loop backbone conformations end-to-end, achieving sub-angstrom accuracy for canonical loops while providing built-in uncertainty estimates critical for prioritizing experimental validation [59,60]. However, like earlier methods, ABlooper’s performance on the conformationally diverse CDR-H3 loop remains limited, underscoring the persistent challenge of modeling this critical antigen-binding region [61]. Specialized approaches have emerged to address this bottleneck: the H3-OPT toolkit integrates AlphaFold2 with a protein language model pre-trained on antibody sequences, reducing CDR-H3 prediction errors to 2.24 Å RMSDαC in benchmark studies—a marked improvement over general-purpose predictors [62]. This advance stems from combining global fold recognition capabilities with sequence–structure relationships learned from structural databases like SAbDab, enabling explicit optimization of H3 loop geometry during prediction [62,63].

Parallel innovations in physics-based modeling, such as the introduction of C-terminal structural constraints derived from conserved kinked conformations, have demonstrated that biasing conformational sampling toward experimentally observed geometries can yield sub-angstrom H3 predictions even in the absence of homologous templates [64,65]. Modern pipelines increasingly combine these approaches, employing deep learning for rapid initial predictions followed by energy minimization protocols (e.g., OpenMM and YASARA) to refine side-chain packing and loop conformations while maintaining computational efficiency [59,66]. For example, H3-OPT’s predictions were experimentally validated through solved nanobody structures, confirming its utility in practical design workflows [62]. Meanwhile, frameworks like IgFold and ABlooper circumvent the speed limitations of Rosetta-dependent tools (e.g., DeepAb, which requires ~10 min per prediction) by leveraging language model embeddings or geometric deep learning to deliver structures in under 25 s with per-residue confidence metrics [60,67]. These advancements collectively establish antibody structure prediction as a multi-resolution problem where sequence-based priors, geometric constraints, and physics-based refinement synergistically overcome the limitations of any single methodology, paving the way for scalable exploration of synthetic antibody repertoires.

In ADC development, the antibody must fulfill more than antigen recognition; it must tolerate site-specific conjugation, maintain structural integrity post-modification, and often promote efficient cellular internalization. The development of ADCs increasingly relies on in silico tools to evaluate critical biophysical properties of antibodies, enabling the rational selection of candidates with optimal stability, specificity, and developability. Computational platforms such as RosettaAntibodyDesign (RAbD) provide a framework for structure-based antibody optimization, leveraging energy minimization and molecular dynamics (MD) simulations to refine antigen-binding interfaces and predict conformational stability [68,69]. For instance, MD simulations have been used to assess the stability of antibody–antigen complexes over 100 ns trajectories, identifying weak binders that dissociate prematurely, thereby guiding affinity maturation efforts [68].

Complementary tools like the Therapeutic Antibody Profiler (TAP) flag developability risks—such as anomalous hydrophobicity, charge asymmetry, or excessive CDR length—by benchmarking against clinical-stage monoclonal antibodies, helping ensure candidates avoid aggregation or immunogenicity [70]. Deep learning platforms such as BioPhi further enhance humanization by evaluating “humanness” scores derived from natural antibody repertoires, thus reducing immunogenic potential while preserving binding efficacy [71]. These computational tools synergize with curated databases like ADCdb, which catalog structural and functional ADC data, enabling cross-validation of predictions against empirical parameters such as linker–payload stability and intracellular trafficking [72]. However, challenges remain in modeling complex molecular recognition events, particularly regarding off-target toxicity or intracellular payload release kinetics—limitations that require iterative integration of computational predictions with experimental validation [64,73].

Significant developments have demonstrated how in silico approaches targeting the antibody component of ADCs have significantly contributed to the development of more effective therapeutics. For instance, Zhao et al. employed molecular dynamics simulations to investigate how antigen binding can allosterically promote Fc receptor recognition, providing critical insights into how structural modifications in the antibody region could enhance effector functions such as antibody-dependent cellular cytotoxicity (ADCC) [74]. Similarly, Kralj et al. utilized MD simulations to explore interactions between IgG1 antibodies and Fc receptors, revealing molecular details that govern binding affinity and specificity. These studies illuminate the mechanistic basis of Fc-mediated immune responses and inform strategies for engineering antibodies with improved effector functions, which are crucial for ADC performance [75].

Beyond receptor interactions, in silico modeling has facilitated rational modification of antibody regions to improve stability, reduce immunogenicity, and optimize conjugation sites. Li et al. demonstrated that site-selective chemoenzymatic modification of the antibody core fucose led to a 3-fold increase in Fcγ receptor IIIa (CD16a) binding affinity and correspondingly enhanced ADCC activity by approximately 2.5-fold. These modifications exemplify how computational predictions can guide antibody engineering to improve effector recruitment in ADC contexts. Furthermore, MD simulations have been applied to evaluate the structural flexibility and conformational stability of antibody variable regions, informing optimal conjugation site selection that preserves antigen binding while enabling efficient payload attachment [76]. The integration of computational tools has become indispensable for addressing the multifaceted requirements of antibody engineering in ADC development, from antigen recognition fidelity to biophysical robustness under therapeutic conditions. These advancements enable systematic prediction of stability, specificity, and manufacturability. However, challenges remain in modeling dynamic intracellular processes such as payload release kinetics, highlighting the need for iterative computational–experimental workflows and experimental validation [77,78]. Given the specific demands of ADC development, the optimization of the linker component is a central focus in ADC design. The linker governs conjugate stability, pharmacokinetics, the therapeutic index, and toxicity.

## 4. Linkers in ADCs

The linker is a critical structural and functional component of ADCs, serving as a covalent bridge between the monoclonal antibody and the cytotoxic payload [19,79]. This bifunctional molecule directly governs ADC stability, pharmacokinetics, and pharmacodynamics by maintaining circulatory integrity during systemic circulation while enabling controlled payload release at target sites, a balance essential for optimizing the therapeutic index. An ideal linker must satisfy three key requirements: (1) prevent ADC aggregation through optimized hydro/lipophilicity, (2) minimize premature payload release in plasma (half-life ≥ 10× ADC circulation time), and (3) facilitate rapid, tumor-specific drug liberation post-internalization [4,79,80].

Modern linker designs fall into two major mechanistic classes: cleavable and non-cleavable. Cleavable linkers exploit physiological gradients (e.g., lysosomal proteases, acidic pH, or intracellular glutathione) for tumor-selective activation, whereas non-cleavable variants require complete antibody degradation in lysosomes to release payloads [4,19,81]. Recent advances highlight the importance of conjugation chemistry and site-specific modifications in fine-tuning linker stability and payload release kinetics, with emerging strategies focusing on optimizing drug–antibody ratios (DARs) and reducing immunogenicity risks [33,73,82]. These developments highlight the linker as a central molecular gatekeeper, critically influencing ADC efficacy, safety, and clinical success [73,79,80].

Cleavable linkers exploit physiological differences between systemic circulation (neutral pH and low reducing potential) and the tumor microenvironment (TME) or intracellular compartments (acidic pH, proteolytic enzymes, and elevated glutathione) to enable precise, context-dependent cytotoxic payload release [4,79,83]. These linkers are generally classified into two subtypes: chemically cleavable linkers, which respond to abiotic triggers such as pH or redox gradients, and enzymatically cleavable linkers, activated by tumor-associated proteases or glucuronidases [4,19,79,83]. Chemically cleavable variants include hydrazone (acid-labile) and disulfide (reduction-sensitive) bonds, whereas enzymatically cleavable designs typically feature peptide bonds (e.g., Val-Cit and Val-Ala) and β-glucuronide linkages. Pyrophosphate-diester linkers, although less common, constitute an emerging class characterized by traceless release and enhanced hydrophilicity [4,9,83,84].

Hydrazone-based linkers leverage the pH gradient between plasma (pH 7.4) and lysosomal/endosomal compartments (pH 4.8–6.2) to trigger hydrolysis. Their circulatory stability combined with rapid cleavage in acidic organelles facilitates targeted payload release, exemplified by the FDA-approved gemtuzumab ozogamicin (Mylotarg^®^) for acute myeloid leukemia. Nonetheless, hydrazones are prone to non-negligible plasma hydrolysis due to their intrinsic chemical lability, leading to premature payload release and dose-limiting toxicity [19,84]. Consequently, their clinical application remains largely restricted to hematologic malignancies, where rapid internalization into acidic compartments mitigates off-target effects [4,19,81].

In contrast, disulfide-based linkers exploit elevated intracellular glutathione (GSH) concentrations (1–10 mM in tumor cells vs. ~2 μM in plasma) to enable reductive cleavage [6,10,85]. Tumor cells frequently upregulate GSH to counter oxidative stress, creating a redox gradient that favors selective payload release post-internalization [6,10]. Although disulfide linkers offer greater plasma stability compared to hydrazones, premature cleavage may occur due to extracellular disulfide isomerases or thiol-containing proteins, necessitating structural optimizations such as steric hindrance or incorporation of hydrophilic spacers [84,85]. Recent innovations, exemplified by trastuzumab emtansine (T-DM1), integrate non-cleavable linkers with lysosomal degradation pathways to circumvent these limitations, albeit at the expense of bystander effects [9,10].

Peptide-based linkers represent a cornerstone of cleavable ADC design due to their reliance on lysosomal proteases for payload release, a mechanism that balances systemic stability with intracellular activation. Among these, valine–citrulline (Val-Cit) and phenylalanine–lysine (Phe-Lys) dipeptide motifs are the most clinically validated, leveraging the enzymatic activity of cathepsin B—a cysteine protease enriched in lysosomes and frequently overexpressed in malignant cells [79,84,85]. While cathepsin B was historically considered the primary mediator of cleavage, recent studies reveal broader susceptibility to other lysosomal cathepsins (e.g., cathepsins K, L, and S), which may contribute to off-target payload release in normal tissues expressing these enzymes [86,87]. Despite this, peptide linkers exhibit superior plasma stability compared to hydrazone or disulfide systems, as their cleavage requires both the acidic lysosomal environment and protease activity, minimizing premature drug release in circulation [79,88].

The Val-Cit-PABC (para-aminobenzyl carbamate) linker exemplifies this class, featuring a self-immolative PABC spacer that enhances enzymatic accessibility to the cleavage site while accommodating bulky payloads like monomethyl auristatin E (MMAE) [84,86]. Structural analyses demonstrate that the PABC moiety undergoes 1,6-elimination post-cleavage, ensuring traceless release of the unmodified payload [85,86]. This design underpins the success of brentuximab vedotin, an anti-CD30 ADC approved for lymphoma, where controlled payload release correlates with reduced systemic toxicity [84,88]. However, Val-Cit-PABC stability can vary across ADC constructs, prompting iterative optimization of spacer chemistry and dipeptide sequences to mitigate hydrolysis or aggregation risks. For instance, cyclobutane-1,1-dicarboxamide (cBu)-based linkers have been engineered to restrict cleavage specificity to cathepsin B, thereby minimizing off-target activation by other proteases [5,87].

Among notable developments, phosphatase-sensitive pyrophosphate diester linkers exploit lysosomal acid phosphatases and pyrophosphatases for payload liberation. These linkers feature a two-step activation mechanism: enzymatic hydrolysis first generates a monophosphate intermediate, followed by spontaneous elimination to release the cytotoxic agent [84,85]. Unlike peptide-based systems, pyrophosphate linkers combine high aqueous solubility—reducing nonspecific cellular uptake—with tunable release kinetics achievable through structural modifications to the diester backbone [85]. Preclinical studies highlight their efficacy in HER2^+^ breast cancer models, where phosphatase-cleavable ADCs outperformed both Val-Cit-PABC-based conjugates and the non-cleavable ADC T-DM1 in tumor suppression [85]. This adaptability positions phosphatase-sensitive systems as promising candidates for ADCs requiring precise spatiotemporal control over payload delivery.

While cleavable linkers enable efficient payload release through enzymatic or chemical triggers, their dependence on tumor-associated proteases or redox gradients introduces variability in heterogeneous malignancies. Furthermore, the bystander effect—a hallmark of cleavable systems like Val-Cit-PABC—can exacerbate toxicity in tumors with mixed antigen expression or leaky microenvironments [82,88]. These limitations have driven the development of non-cleavable linkers, which forgo enzymatic activation and instead rely on complete antibody degradation in lysosomes to release payloads conjugated to residual amino acids (e.g., lysine or cysteine). By eliminating premature cleavage risks and restricting payload release to fully internalized ADCs, non-cleavable systems offer enhanced plasma stability and reduced off-target effects, albeit at the cost of requiring highly potent payloads and antigen-specific internalization [82,89,90].

Cleavable linkers, while advantageous for enabling payload release under specific intracellular conditions, are often limited by reduced plasma stability and a higher propensity for off-target effects. In contrast, non-cleavable linkers offer a strategic alternative by ensuring that the cytotoxic payload is released only after complete internalization of ADCs and its subsequent degradation within the lysosomes of the target cell. These linkers employ chemically robust, non-reducible bonds—most commonly thioethers—that are resistant to the proteolytic cleavage, acidic pH, and reductive environments encountered during systemic circulation and early endosomal processing [9,85,91]. As a result, non-cleavable ADCs demonstrate significantly improved plasma stability, thereby minimizing premature drug release and systemic toxicity [9,31]. The mechanism of action is wholly dependent on the intracellular catabolism of the monoclonal antibody, which ultimately liberates the payload conjugated to a residual amino acid (e.g., lysine or cysteine), forming a charged drug-linker–amino acid complex. Owing to its hydrophilic character, this metabolite exhibits poor membrane permeability, confining its cytotoxic action to the intracellular environment of antigen-positive cells and reducing bystander effects on neighboring antigen-negative cells [91,92]. While the efficacy of this approach depends on the use of highly potent payloads and efficient antigen-mediated internalization, it is particularly advantageous in the context of tumors with heterogeneous antigen expression or in clinical settings where bystander toxicity poses a significant risk [9,93].

T-DM1, an FDA-approved ADC for HER2-positive breast cancer, exemplifies the successful clinical translation of a non-cleavable linker strategy. T-DM1 employs the thioether linker succinimidyl-4-(N-maleimidomethyl) cyclohexane-1-carboxylate (SMCC) to conjugate the maytansinoid derivative DM1 to the anti-HER2 antibody trastuzumab [92]. Its therapeutic efficacy hinges on sequential intracellular processing: (1) specific binding to HER2 receptors on tumor cells, (2) internalization of the ADC–receptor complex, (3) trafficking to lysosomes, (4) complete proteolytic degradation of the trastuzumab antibody, and (5) release of the active metabolite, lysine-MCC-DM1 (Lys-MCC-DM1) [91,92]. The charged nature of Lys-MCC-DM1, a direct consequence of the non-cleavable linker design and the retention of the linker plus a lysine residue from the antibody, impedes its diffusion across cellular membranes [92,94]. This characteristic ensures cytotoxic activity is primarily restricted to HER2-positive cells that have internalized the ADC, significantly reducing off-target effects compared to ADCs utilizing cleavable linkers capable of generating membrane-permeable payloads [9,73,85]. Although preclinical studies suggest newer cleavable linker systems, such as phosphatase-sensitive pyrophosphate diesters, may demonstrate superior potency in certain HER2^+^ models compared to T-DM1 [4,85], T-DM1’s clinical validation underscores the effectiveness and safety achievable with optimized non-cleavable linker technology for targeted intracellular drug release.

Recent innovations in linker technology focus on enhancing plasma stability, payload release precision, and therapeutic versatility. Hydrophilic modifications represent a key strategy to address aggregation and pharmacokinetic challenges. For instance, structural optimization of linkers through polyethylene glycol (PEG) integration or charged moieties reduces hydrophobicity in high-drug–antibody-ratio ADCs, mitigating aggregation and rapid clearance while maintaining plasma stability [95]. These modifications are particularly valuable for site-specific conjugates, where controlled hydrophilicity balances payload delivery and systemic exposure. Additionally, tandem cleavage systems—an innovation in cleavable linker design—engineer dual enzymatic triggers (e.g., glucuronidase followed by cathepsin) that sequentially activate only within lysosomes [96]. This dual-step mechanism prevents premature release in circulation while ensuring efficient payload liberation intracellularly, improving both plasma stability (>1 week half-life) and tolerability in preclinical models [31,96].

Site-specific conjugation further revolutionizes ADC design by enabling homogeneous DAR distribution through engineered cysteine residues or bioorthogonal chemistry. Unlike stochastic conjugation, this approach eliminates heterogeneous populations that cause variable pharmacokinetics and off-target toxicity [97]. For example, pyrophosphate-diester linkers conjugated site-specifically to glucocorticoid payloads demonstrate enhanced stability and potency, underscoring how spatial control over linker attachment optimizes drug release kinetics and reduces aggregation [98]. These advancements collectively expand ADC applicability to tumors with variable antigen density or stromal barriers, where controlled bystander effects or enhanced penetration are critical [10].

Recent innovations in linker architecture, such as Exo-linkers and tandem linkers, address the limitations of traditional Val-Cit-PABC and SMCC linkers by improving stability, controlling payload release, and expanding the therapeutic window. Exo-linkers represent an innovative advancement in ADC linker chemistry by repositioning the traditional cleavable peptide linker, such as Val-Cit, to the exo-position of the PAB moiety. This structural repositioning enhances the enzymatic and plasma stability of the linker–payload connection, significantly reducing premature payload release and hydrophobicity-induced aggregation—a major limitation of conventional Val-Cit linkers. For example, mouse plasma studies have shown that free payload concentrations remain below 5% after four days of incubation, underlining the remarkable resistance of Exo-linkers to premature cleavage. The introduction of hydrophilic residues, such as glutamic acid, into the Exo-linker backbone improves solubility, allowing ADCs to maintain higher DARs without aggregation-related manufacturability issues. Indeed, preclinical data demonstrate that Exo-linker-based ADCs can achieve DARs greater than 8, compared to the typical DAR range of 3–4 observed with traditional Val-Cit linkers, while preserving conjugate stability even in the presence of destabilizing enzymes like carboxylesterases and neutrophil elastase [99].

Hydrophobic interaction chromatography confirmed their improved hydrophilicity compared to traditional Val-Cit linkers, reducing aggregation and enhancing pharmacokinetics. Unlike Val-Cit linkers, which are susceptible to neutrophil elastase (NE)-mediated cleavage leading to premature payload release and off-target toxicity, Exo-linkers remained intact under NE exposure, preventing unwanted drug liberation. These properties collectively demonstrate that Exo-linkers address key limitations of conventional linkers, offering enhanced stability, safety, and therapeutic reliability for ADC development [100]. Tandem-cleavage linkers represent a novel design in ADCs that improve in vivo stability and reduce systemic toxicity by requiring two sequential enzymatic cleavage steps to release the cytotoxic payload. The first cleavage is typically performed by β-glucuronidase, an enzyme highly expressed in tumor lysosomes and the tumor microenvironment, which removes a glucuronide protecting group attached to the linker. This enzymatic removal exposes a secondary peptide bond, which is subsequently cleaved by intracellular proteases, releasing the active payload specifically inside target cells. This dual enzymatic activation mechanism minimizes premature payload release in circulation, thereby enhancing plasma stability and significantly lowering off-target toxicities, such as the myelosuppression commonly observed with traditional cleavable linkers [101].

Compared to conventional Val-Cit linkers that rely on a single protease cleavage event—usually by cathepsin B—tandem-cleavage linkers incorporate an additional protective glucuronide moiety that sterically hinders extracellular enzyme-mediated cleavage. This design markedly decreases the nonspecific payload release seen in Val-Cit linkers, which are susceptible to cleavage by multiple proteases including serine elastase, contributing to systemic toxicities. In preclinical models of CD79b-targeted ADCs, tandem-cleavage linkers demonstrated substantially improved in vivo stability and tolerability, with reduced myelosuppression relative to Val-Cit linked ADCs. By more precisely controlling drug release through sequential cleavage, tandem-cleavage linkers enhance the therapeutic window and manufacturability of ADCs, supporting more stable payload retention and sustained antitumor efficacy [101].

Computational methodologies are increasingly pivotal in advancing ADC linker design, though current sources emphasize conceptual foundations rather than specific tools. In silico strategies, particularly molecular dynamics simulations, model linker–antibody interactions to predict stability under physiological conditions (e.g., proteolytic or acidic environments). Such simulations assess conformational flexibility and bond susceptibility, guiding the design of non-cleavable linkers resistant to serum proteases or cleavable linkers optimized for tumor-specific enzyme recognition (e.g., legumain or cathepsin B) [86]. Machine learning algorithms further accelerate this process by analyzing structure–activity relationships from historical ADC data, forecasting the plasma stability and cytotoxicity of novel linker–payload metabolites [102]. For instance, predictive models can identify amino acid–linker configurations that balance hydrophilicity (to limit bystander effects) and potency, as seen in non-cleavable linker catabolites like lysine-MCC-DM1 [85,102].

Beyond these conceptual strategies, practical applications demonstrate how in silico methods are transforming ADC development. A notable example is the Phase III anti-TROP2 ADC SKB264, where the ADCNet platform employed AI-driven screening of over 1200 linker candidates to identify a PEG-based, dual pH/enzyme-responsive linker optimized for circulatory stability and tumor-selective payload release. This design extended the plasma half-life by ~2.3-fold and improved tumor-to-plasma selectivity from 5:1 to 18:1 compared to conventional linkers [103]. These results show how predictive modeling and machine learning can accelerate linker design and deliver clinically relevant improvements in ADC performance.

In parallel with these applied successes, emerging generative models such as Linker-GPT illustrate the next frontier in computational linker design. This framework leverages chemical language models and energy-based scoring functions to generate and evaluate novel linker candidates, optimizing for stability, cleavability, and synthetic feasibility. By enabling rapid virtual screening of thousands of designs, Linker-GPT reduces experimental cycles and enhances early-stage decision-making, offering a powerful complement to traditional molecular dynamics and SAR-driven approaches [104].

Future development will likely integrate multi-scale computational platforms, combining quantum mechanical calculations for bond stability with systems pharmacology models to simulate patient-specific ADC trafficking and payload release. Such tools could optimize tandem enzymatic triggers or site-specific conjugation sites in silico, reducing empirical screening. However, experimental validation remains essential, as underscored by studies highlighting the context-dependent nature of linker performance (e.g., antigen internalization efficiency and lysosomal protease variability) [10,19]. As computational pipelines mature, they will enable rational design of linkers tailored to tumor microenvironmental cues, ultimately expanding the therapeutic index of next-generation ADCs.

## 5. Payloads in ADCs

The therapeutic efficacy of antibody–drug conjugates is fundamentally constrained by the limited fraction of the administered dose that ultimately reaches the tumor site. This inefficient delivery necessitates payloads with exceptionally high cytotoxic potency to achieve meaningful tumor cell eradication. As highlighted in clinical and preclinical analyses, effective payloads must eliminate most target cells even at extremely low concentrations, typically in the nanomolar to picomolar range for their half-maximal inhibitory concentration values [82]. This requirement stems from the complex pharmacokinetics of ADCs, where only a small proportion of the injected conjugate successfully internalizes into target cells, while the remainder circulates systemically or distributes to healthy tissues. Consequently, payloads with marginal potency fail to achieve sufficient tumor cell killing at achievable doses. Furthermore, the high potency enables the use of lower DARs, which is critical for maintaining antibody stability and minimizing premature payload release during circulation. This balance is essential, as excessively high DARs can promote antibody aggregation, accelerate clearance, and increase off-target toxicity, thereby undermining therapeutic efficacy [31,82,90]. The clinical imperative for ultra-potent payloads is further underscored by the high failure rates of early ADCs employing less cytotoxic chemotherapeutics like doxorubicin or vinblastine, which demonstrated limited antitumor activity [82].

An ideal ADC payload must possess a stringent combination of biochemical and physicochemical properties to ensure therapeutic success. Paramount among these is high systemic stability. Payloads must remain inert and intact during circulation within the bloodstream, resisting degradation prior to reaching the target site. Instability during conjugation, storage, or in vivo transit leads to premature payload release, causing systemic toxicity and reducing the effective dose delivered to the tumor [31,82]. Efficient and stable antibody conjugation is equally critical. The payload must contain modifiable functional groups (e.g., amines, thiols, and carboxylic acids) compatible with robust conjugation chemistries to form stable linkages (via the linker) to the antibody. The conjugation process itself must not compromise the payload’s potency or the antibody’s integrity and binding capability [90,105]. Furthermore, payloads must exhibit low immunogenicity to avoid eliciting neutralizing anti-drug antibodies that could accelerate ADC clearance and reduce efficacy or cause adverse immune reactions [105]. High solubility or adequate formulation compatibility is essential to prevent aggregation during manufacturing, storage, or administration, which can alter pharmacokinetics and increase toxicity risks [82]. Crucially, the payload must maintain its potent cytotoxic activity once released intracellularly after internalization and linker cleavage. Achieving this complex interplay of properties—ultra-high potency, stability, conjugation compatibility, low immunogenicity, and solubility—remains a central challenge in ADC design, directly impacting the therapeutic index and clinical viability of the conjugate [6,82,90,105].

Payloads integrated into ADCs are broadly classified into four principal categories based on their mechanism of action and prevalence in clinical development. Tubulin inhibitors, such as auristatins (e.g., monomethyl auristatin E and MMAE) and maytansinoids (e.g., DM1 and DM4), constitute one major class. These agents disrupt microtubule dynamics during cell division, inducing mitotic arrest and apoptosis in rapidly proliferating cancer cells. DNA-damaging agents represent another dominant category, encompassing payloads like calicheamicins and duocarmycins. These compounds induce double-strand DNA breaks or alkylate DNA, triggering catastrophic genomic damage and cell death [6,105]. Topoisomerase I inhibitors, exemplified by exatecan derivatives (e.g., DXd), have emerged as a highly successful third class. These inhibitors stabilize the topoisomerase I–DNA cleavage complex, preventing DNA religation and generating lethal replication-associated DNA damage [6].

### 5.1. Tubulin Inhibitors

Tubulin inhibitors represent a cornerstone class of payloads in ADCs, leveraging their potent cytotoxicity to selectively target tumor cells while minimizing systemic exposure. These agents disrupt microtubule dynamics, essential for mitosis, intracellular transport, and cell motility, leading to cell cycle arrest and apoptosis. In ADCs, tubulin inhibitors are conjugated via cleavable or non-cleavable linkers to monoclonal antibodies that recognize tumor-specific antigens. Upon internalization and linker cleavage, the payload is released intracellularly, exerting cytotoxic effects. Notably, tubulin inhibitors exhibit bystander-killing capabilities, where released payloads diffuse to adjacent cells, enhancing efficacy in heterogeneous tumors [105]. However, their efficacy is constrained by preferential activity against rapidly dividing cells, potentially sparing quiescent tumor populations and necessitating higher dosing regimens compared to payloads like anthracyclines [12].

The primary chemical classes of tubulin inhibitors employed as ADC payloads include auristatins, maytansinoids, and tubulysins, each characterized by distinct mechanisms and potencies. Auristatins, such as monomethyl auristatin E (MMAE), bind the β-subunit of tubulin dimers, promoting aberrant polymerization and destabilizing microtubule dynamics, with half-maximal inhibitory concentrations ranging from 0.05 to 0.1 nM [106]. Clinically approved ADCs utilizing auristatins include brentuximab vedotin (MMAE conjugate), used in Hodgkin lymphoma and anaplastic large-cell lymphoma [107]. Maytansinoids, exemplified by DM1 (mertansine), inhibit tubulin polymerization by binding to the vinblastine site, preventing microtubule assembly (IC50: 0.05–0.1 nM) [108]. T-DM1, approved for HER2-positive breast cancer, is a prominent maytansinoid-based ADC. Tubulysins, a newer class, suppress tubulin polymerization with IC50 values of 0.1–1 nM [109,110]. Though not yet in approved ADCs, tubulysins are under clinical investigation due to their potency against multidrug-resistant tumors [90,111].

Recent innovations in tubulin-inhibitor payloads focus on enhancing metabolic stability, solubility, and tumor selectivity to overcome limitations such as off-target toxicity and drug resistance. For tubulysins, structural optimizations—such as carbamate-containing analogs—improve plasma stability and reduce hepatic metabolism, thereby increasing therapeutic indices [90]. Leverett et al. synthesized tubulysin derivatives with modified macrocycles, demonstrating superior cytotoxicity in heterogeneous tumor models and resistance phenotypes. Similarly, cryptophycin-based payloads, which inhibit tubulin polymerization at picomolar concentrations, are being explored for their efficacy in eradicating low-proliferation tumors, addressing a key weakness of conventional tubulin inhibitors [111]. These advances align with the broader pursuit of next-generation payloads that exhibit higher potency, reduced systemic toxicity, and activity against quiescent cells [5,81].

Clinical findings depict the context-dependent efficacy of tubulin-inhibitor ADCs. They demonstrate pronounced activity in hematological malignancies (e.g., lymphomas) and solid tumors with high mitotic rates, such as breast and urothelial cancers [105,112]. For instance, ADCs like polatuzumab vedotin (an MMAE conjugate) show robust responses in diffuse large B-cell lymphoma, capitalizing on rapid cell division in these tumors [89]. However, in slow-proliferating or stroma-rich solid tumors, efficacy is often limited, necessitating combination therapies or novel payload designs. Recent studies highlight that tubulin inhibitors require higher cumulative doses (>5 mg/kg) than anthracycline payloads to achieve cures in preclinical solid-tumor models, partly due to their reduced activity against quiescent cells [12,113].

### 5.2. DNA-Damaging Agents

DNA-damaging payloads encompass several structurally and mechanistically diverse subclasses, including calicheamicins, duocarmycins, topoisomerase inhibitors, and DNA cross-linkers. Calicheamicins, derived from the soil bacterium *Micromonospora echinospora*, are enediyne antibiotics that induce sequence-specific double-strand DNA breaks via radical-mediated cleavage upon reduction in the cellular environment [5]. This mechanism underlies the efficacy of gemtuzumab ozogamicin, an anti-CD33 ADC approved for acute myeloid leukemia (AML), which delivers N-acetyl-γ-calicheamicin dimethyl hydrazide with an IC50 of 10–50 pM in leukemia cell lines [114]. Duocarmycins, synthetic analogs of natural products from Streptomyces species, function as DNA minor groove alkylating agents, forming covalent adducts that disrupt replication and transcription. While no duocarmycin-based ADCs are yet approved, investigational agents like SYD985 (trastuzumab-duocarmycin) have demonstrated potent antitumor activity in HER2-positive cancers, with preclinical IC50 values below 100 pM, though clinical development has faced challenges related to metabolic instability [5,72]. Topoisomerase I inhibitors, exemplified by the exatecan derivative DXd in trastuzumab deruxtecan, trap topoisomerase I–DNA cleavage complexes during DNA relaxation, causing lethal replication fork collapse [5,8]. Approved for HER2-positive breast and gastric cancers, trastuzumab deruxtecan achieves high cytotoxic potency (IC50 in the low nanomolar range) and induces significant bystander killing, enabling activity even in tumors with moderate or low HER2 expression [115]. DNA cross-linking agents, such as pyrrolobenzodiazepines (PBDs) and novel synthetic compounds, form inter-strand cross-links that block DNA separation, with preclinical ADCs (e.g., those employing SG3199) exhibiting IC50 values of 20–50 pM in lymphoma models [72,91,116].

Clinically, DNA-damaging payloads excel in contexts where tubulin inhibitors show reduced efficacy, particularly in hematologic malignancies with high DNA repair deficiencies (e.g., AML via gemtuzumab ozogamicin) and solid tumors exhibiting low or heterogeneous target antigen expression [5,83]. The bystander effect of payloads like DXd enables trastuzumab deruxtecan to effectively target HER2-heterogeneous breast cancers, achieving objective response rates of up to 70% in pretreated HER2-positive disease and over 50% in HER2-low tumors [117]. In contrast, tubulin inhibitors often require homogeneous, high-level antigen expression for optimal activity, limiting utility in antigenically diverse tumors [5,11]. Emerging data also support DNA-damaging ADCs in minimal residual disease settings due to their ability to eradicate quiescent cancer cells, a niche where mitotic inhibitors underperform [5,6].

Resistance to DNA-damaging payloads in ADCs can arises from several biological mechanisms that compromise therapeutic efficacy. Enhanced DNA repair pathways, particularly homologous recombination (HR) and non-homologous end joining (NHEJ), enable cancer cells to effectively resolve DNA lesions induced by ADC payloads, thereby mitigating cytotoxic effects. Overexpression or upregulation of key DNA repair proteins such as RAD51 (in HR) and DNA-PKcs (in NHEJ) has been implicated in resistance, facilitating efficient repair of double-strand breaks and cross-links [118,119]. Concurrently, increased activity of efflux transporters, notably members of the ATP-binding cassette (ABC) family such as P-glycoprotein (MDR1), actively pumps payload molecules out of tumor cells, reducing intracellular concentrations of cytotoxic agents and limiting their potency. Alterations in apoptotic signaling pathways further contribute to resistance by impeding programmed cell death; dysregulation of pro-apoptotic factors (e.g., BAX and BAK) or overexpression of anti-apoptotic proteins (e.g., BCL-2 and MCL-1) shifts the balance toward cell survival despite DNA damage [120,121]. To counteract these resistance mechanisms, combination therapies incorporating PARP inhibitors have gained prominence, exploiting synthetic lethality by inhibiting single-strand break repair and thus overwhelming the cancer cell’s DNA repair capacity. Modifications to payload chemistry are also under investigation to enhance payload retention and evade efflux, including the development of dual-payload ADCs that leverage complementary mechanisms to overcome heterogeneous resistance. Additionally, strategies targeting resistant tumor subclones through biomarker-guided ADC design and combination regimens aim to prevent or delay resistance emergence. These approaches collectively seek to restore ADC efficacy by circumventing DNA repair proficiency, efflux-mediated drug clearance, and apoptotic resistance associated with DNA-damaging payload resistance [122,123].

Despite their potential, DNA-damaging ADCs face challenges related to their extreme potency, which increases the risk of on-target toxicity in normal tissues expressing low levels of the antigen, exemplified by the hepatic veno-occlusive disease observed with gemtuzumab ozogamicin [4]. Premature payload release due to instability during systemic circulation can exacerbate toxicities such as myelosuppression and gastrointestinal damage, while suboptimal linker stability may compromise tumor-selective payload delivery. Maintaining a narrow therapeutic window necessitates rigorous optimization of drug-to-antibody ratios and conjugation sites to maximize efficacy and minimize adverse effects. Future improvements will depend on innovations in linker chemistry—such as protease-cleavable tetrapeptides—tumor-selective payload activation, and refined payload engineering to limit systemic exposure [72,87]. The integration of DNA-damaging ADCs with multimodal approaches, including radioimmunoconjugates and PARP inhibitors, offers significant potential for exploiting DNA repair deficiencies, notably in BRCA-mutated cancers. As payload diversity advances, DNA-damaging agents will continue to play a pivotal role in extending ADCs’ therapeutic reach to resistant and refractory malignancies, provided that translational strategies effectively mitigate associated toxicological risks.

### 5.3. Emerging Agents

Recent advances in ADC technology have spurred the exploration of novel payload classes beyond traditional microtubule inhibitors and DNA-damaging agents. These emerging payloads aim to address limitations such as drug resistance, off-target toxicity, and limited efficacy in immunologically “cold” tumors. By leveraging diverse mechanisms—including immune modulation, transcriptional interference, and metabolic disruption—these new agents offer the potential to expand the therapeutic scope of ADCs and improve clinical outcomes across a broader range of cancer types. The following sections highlight key categories of these next-generation payloads, emphasizing their mechanisms of action, design challenges, and current preclinical or clinical progress [5,6,105].

Dual-payload ADCs represent a promising advancement in targeted cancer therapy by incorporating two cytotoxic agents with distinct mechanisms of action into a single construct. This dual approach enhances therapeutic efficacy through synergistic effects, such as combining DNA-damaging agents with PARP inhibitors or microtubule inhibitors with immunomodulators, allowing simultaneous targeting of multiple pathways and immune activation. Moreover, the mechanism complementarity of dual payloads helps overcome drug resistance, as tumor cells resistant to one agent may still respond to the other. The ability to fine-tune drug-to-antibody ratios (e.g., 2 + 2 or 4 + 2) further optimizes the therapeutic window by reducing systemic toxicity while maintaining potency. Emerging designs also exploit the bystander effect to remodel the tumor microenvironment. Since the concept was first introduced in 2017 by Levengood et al. [124] with MMAE/MMAF-based ADCs, numerous dual-payload formats have been developed, including combinations with PBD dimers and RNA polymerase II inhibitors. Recent advances in site-specific conjugation techniques, such as click chemistry, have facilitated the generation of more homogeneous and flexible dual-payload constructs. Several novel dual-payload ADCs have since entered preclinical and clinical development, targeting antigens such as HER2, Trop2, and EGFR, and incorporating diverse payload combinations [125,126]. Among them, KH815—an ADC targeting Trop2 with a topoisomerase I and RNA polymerase II inhibitor payload—was the first dual-payload candidate to reach clinical trials, demonstrating robust preclinical antitumor activity, resistance circumvention, and a favorable safety profile [127].

Proteolysis Targeting Chimeras (PROTACs) as ADC payloads represent a cutting-edge strategy combining the catalytic protein degradation mechanism of PROTACs with the tissue specificity of antibody delivery. PROTAC molecules are heterobifunctional compounds that recruit E3 ubiquitin ligases to tag specific intracellular proteins for proteasomal degradation, thereby modulating protein levels with catalytic efficiency at low doses. When conjugated to antibodies, PROTAC payloads enable targeted delivery of these degraders to cancer cells expressing the ADC’s surface antigen. Upon internalization and lysosomal processing of the ADC, the active PROTAC is released intracellularly, where it induces selective degradation of oncogenic or disease-causing proteins. This targeted mechanism expands ADC payload functionality beyond classic cytotoxicity, offering the potential to degrade “undruggable” intracellular targets. Furthermore, PROTAC conjugation has been shown to enhance antibody internalization and improve cytotoxicity in vitro. Antibody–PROTAC conjugates can overcome limitations inherent to both modalities by combining the catalytic potency and broad target range of PROTACs with the selective delivery and favorable pharmacokinetics of antibodies, making them a promising platform for next-generation targeted therapies. This approach has demonstrated robust antigen-dependent protein degradation and tumor regression in preclinical models, paving the way for improved efficacy and reduced off-target toxicity in clinical applications [128,129,130].

Antibody–oligonucleotide conjugates (AOCs), representing a cutting-edge RNA-targeting payload format, enable precise delivery of oligonucleotides such as siRNA or antisense oligonucleotides to specific cells via antibody targeting. These conjugates combine the cell-specific binding and internalization capability of antibodies with the gene-silencing or gene-regulatory potential of RNA-based therapeutics. By conjugating oligonucleotides to antibodies directed against tumor- or disease-relevant antigens, AOCs facilitate targeted modulation of gene expression in affected cells, expanding the therapeutic utility of ADC-like strategies beyond classic cytotoxicity into the regulation of intracellular pathways at the RNA level. AOCs help overcome key delivery challenges associated with naked oligonucleotides, including poor cellular uptake and off-target effects, thereby improving the therapeutic index. Recent research highlights progress in linker chemistries and conjugation methods optimized to preserve oligonucleotide stability and activity, as well as in vivo efficacy of antibody–siRNA conjugates in relevant disease models. This innovative payload modality broadens the scope of antibody conjugates, offering new avenues for treatments in oncology and beyond by enabling targeted RNA interference with high specificity and reduced systemic toxicity [131,132,133].

NAMPT inhibitor payloads represent a novel and promising class of ADC payloads that exploit the inhibition of nicotinamide phosphoribosyltransferase (NAMPT), a key enzyme involved in intracellular NAD+ biosynthesis critical for cellular metabolism and survival. Unlike classical cytotoxic agents targeting mitosis or DNA, NAMPT inhibitors induce tumor cell death by depleting NAD+ levels, leading to metabolic catastrophe and apoptosis, with activity observed even in slowly proliferating cancer cells. Recent structure-guided drug design efforts have optimized NAMPT inhibitor payloads to include suitable linker attachment sites, enabling stable conjugation to antibodies targeting various tumor antigens such as HER2, C4.4a, and B7H3 [134]. These NAMPTi-ADCs demonstrate potent, antigen-dependent cytotoxicity in vitro and robust tumor growth inhibition in multiple in vivo xenograft models while maintaining favorable selectivity profiles compared to isotype controls. Optimizations in scaffold chemistry and linker design have also improved payload stability, reduced non-specific toxicity, and mitigated premature payload release. Additionally, for the first time, NAMPTi payload metabolites generated intracellularly from ADC processing have been characterized both in vitro and in vivo, enhancing understanding of their pharmacology and safety. This payload class offers a significant therapeutic advantage by targeting metabolic vulnerabilities in cancer cells and broadening the effectiveness of ADCs beyond the classical paradigm of highly cytotoxic chemotherapy, making NAMPT inhibitors an attractive payload option for next-generation ADC development in both solid and hematological malignancies [134,135,136].

STING (Stimulator of Interferon Genes) agonist payloads represent an emerging and innovative class of ADC payloads that combine direct immune activation with targeted delivery. Upon release inside the tumor microenvironment, these payloads activate the STING pathway in tumor and immune cells, triggering a signaling cascade that culminates in the phosphorylation of TBK1 and IRF3, resulting in robust type I interferon production and pro-inflammatory cytokine secretion. This immune stimulation facilitates dendritic cell maturation, T-cell priming, and recruitment of cytotoxic lymphocytes, effectively converting immunologically “cold” tumors into “hot” ones with enhanced antitumor immunity. Unlike classical cytotoxic payloads, STING agonist payloads primarily act by modulating the tumor immune milieu rather than directly killing tumor cells, reducing the need for a bystander effect. To optimize the therapeutic index, these payloads are engineered to be potent yet hydrophilic, exhibiting low passive membrane permeability to restrict off-target uptake and enable rapid clearance if prematurely released systemically. Careful design of the payload, linker, and scaffold components ensures stability in circulation, efficient release upon ADC internalization, and favorable pharmacokinetics. Preclinical studies have demonstrated that STING agonist ADCs achieve strong tumor growth inhibition with minimal systemic immune activation and can synergize with immune checkpoint inhibitors, highlighting their potential to enhance efficacy while mitigating toxicities seen with free STING agonists. This targeted approach addresses previous clinical limitations of small-molecule STING agonists and represents a promising advancement in immunomodulatory ADC development [137,138,139].

## 6. Quantitative Framework for Optimizing Antibody–Drug Conjugates

The evolution of payload design in ADCs is increasingly driven by sophisticated in silico methodologies, which enhance precision, efficiency, and innovation beyond conventional experimental approaches. Structure-based drug design (SBDD) leverages molecular modeling to optimize linker–payload stability and tumor-specific cleavage efficiency by simulating antibody–payload interactions and conformational dynamics [59,90]. Pharmacophore modeling and molecular docking further refine payload specificity and potency, enabling the rational design of novel immunomodulatory or dual-function agents through virtual screening of compound libraries [90].

While advances in computational linker design, exemplified by frameworks like Linker-GPT [104], promise accelerated optimization and rational tuning of linker properties, the complexity of ADC efficacy demands integration with robust experimental validation. Quantitative, experimentally derived parameters—such as optimal payload lipophilicity, antigen density thresholds, linker cleavage kinetics, the bystander effect radius, and drug-to-antibody ratio stability—provide essential design benchmarks. These empirically established metrics ensure that linker innovations translate into meaningful improvements in the payload delivery, tumor specificity, and therapeutic index in vivo and future parameters for new software.

Multiple studies have established a positive correlation between payload lipophilicity, typically measured by calculated LogD (cLogD), and the efficacy of bystander cell killing in ADCs. Specifically, payloads with cLogD values greater than 2.0, such as exatecan derivatives and MMAE, exhibit enhanced membrane permeability that facilitates diffusion into adjacent antigen-negative tumor cells, generating a robust bystander killing effect in heterogeneous tumor microenvironments [140]. For example, a HER2-targeting ADC conjugated with a novel exatecan payload (Ed9, cLogD > 2) demonstrated efficient tumor bystander killing in both in vitro studies and murine xenograft models with heterogeneous HER2 expression [141]. Although specific quantitative permeability data from assays such as PAMPA are less frequently reported, these payloads consistently induce strong bystander effects in vivo, supporting the role of lipophilicity in promoting payload release and cell membrane permeability necessary for effective bystander killing [140,141,142]

Experimental evidence from xenograft models, patient-derived xenografts, and clinical biomarker studies consistently indicates that effective ADC targeting requires a minimum tumor antigen density typically exceeding 10^5^ receptors per cell, although efficacious ranges have been reported between 5000 and 1,000,000 antigens per cell. These thresholds, quantified primarily by flow cytometry-based characterization of cell lines and tumor samples, are critical for ensuring sufficient ADC binding and internalization to achieve tumor cell killing [96,143]. While higher antigen density generally correlates with increased ADC uptake, an overly strong antibody affinity can lead to the “binding site barrier” effect, limiting tumor penetration due to excessive perivascular binding. To mitigate this, current ADC design strategies employ antibody engineering or utilize smaller antibody formats such as scFvs and nanobodies to optimize the balance between affinity and tissue penetration, thus enhancing therapeutic efficacy in solid tumors [144].

The optimal lysosomal cleavage half-life (t½) for cleavable ADC linkers is generally designed to balance rapid intracellular payload release with stable circulation. Common linkers such as valine–citrulline (Val-Cit) and GGFG peptides have been extensively characterized through in vitro studies using human lysosomal enzymes and in vivo pharmacokinetic analyses, typically achieving efficient payload release with over 85% cleavage within 2 to 4 h under lysosomal conditions while maintaining high plasma stability to prevent premature release. This balance is crucial, with Val-Cit linkers showing enzymatic half-lives around 2.8 to 4.6 h [85].

Quantitative assessment of the bystander effect in three-dimensional cell cultures and primary human tumor xenograft models demonstrates that highly lipophilic payloads (DGN549, cLogD ~4.12) can induce cytotoxic effects in antigen-negative tumor cells located up to 100–200 μm from antigen-positive cells. This distance, equivalent to 10–20 cell diameters, was validated using pharmacodynamic mapping of γH2AX in tumor spheroids and xenograft sections, revealing the payload’s ability to diffuse and maintain lethal concentrations despite heterogeneous antigen expression. The spatial extent of bystander killing is influenced by payload lipophilicity, which balances tissue penetration with cellular uptake to minimize washout [145]. These findings underscore the importance of payload physicochemical properties in ADC design, particularly for targets with moderate or patchy expression patterns, as they can significantly enhance efficacy by compensating for delivery limitations and antigen heterogeneity. However, as data in this area remain relatively limited, further studies across diverse models and payloads are needed to fully define the parameters governing bystander diffusion and the cytotoxic radius for different ADC platforms.

Extensive preclinical and clinical evidence indicates that DARs in the range of two to four generally offer the best balance between therapeutic potency, pharmacokinetic stability, and safety. While higher DARs (>4) may increase the cytotoxic payload delivered to tumor cells, they are often associated with reduced stability, faster systemic clearance, and a higher risk of off-target toxicity due to drug accumulation in healthy tissues. Recent advances in conjugation chemistry—particularly site-specific methods and the use of hydrophilic linkers—have allowed the development of ADCs with higher DARs (up to eight) that still maintain acceptable pharmacokinetic and safety profiles. These optimized constructs preserve structural integrity while delivering effective payload doses, as demonstrated in xenograft models and clinical trials [145,146].

The quantitative parameters discussed above apply differently across ADC architectures. Internalizing ADCs demonstrate superior potency for targets with rapid endocytosis rates, while non-internalizing ADCs require carefully optimized protease-cleavable linkers and highly permeable payloads to achieve efficacy through extracellular activation. Smaller antibody formats (nanobodies and scFvs) exhibit distinct pharmacokinetic profiles characterized by rapid tumor penetration and clearance, requiring adjustment of both DAR and dosing schedules. These format-specific considerations underscore the importance of tailoring ADC design to both target biology and therapeutic context [42,147].

## 7. Future Perspectives

The evolution of payload design in ADCs is increasingly driven by sophisticated in silico methodologies, which enhance precision, efficiency, and innovation beyond conventional experimental approaches. Structure-based drug design leverages molecular modeling to optimize linker–payload stability and tumor-specific cleavage efficiency by simulating antibody–payload interactions and conformational dynamics [59,90]. Pharmacophore modeling and molecular docking further refine payload specificity and potency, enabling the rational design of novel immunomodulatory or dual-function agents through virtual screening of compound libraries [90].

While traditional computational affinity maturation faces challenges in achieving higher than 10-to-100-fold improvements compared to experimental mutagenesis [148], emerging artificial intelligence (AI) techniques overcome these limitations. Deep learning models rapidly predict high-resolution antibody structures and paratope–epitope interactions, guiding targeted mutations to enhance binding affinity and developability [149,150]. Generative AI accelerates de novo payload discovery by creating novel antibody scaffolds and optimizing physicochemical properties—such as hydrophobicity and polyreactivity—to reduce clinical attrition [65,150,151]. Integrative bioinformatics pipelines incorporate multi-omics data and tumor target prediction to prioritize payloads with optimal selectivity and ADMET profiles, using in silico surrogate metrics for early de-risking [152,153].

Current constraints include the limited reliability of free-energy estimations, insufficient training data for specialized applications like nanobody engineering, and validation gaps in generative model outputs [59,148,149]. Future advancements will require community benchmarking, hybrid experimental–computational workflows, and expanded datasets to refine predictive accuracy. Over the next decade, the integration of multi-scale simulations, generative AI for synthesis planning, and zero-shot computational design promises to eliminate dependency on large-scale library screening, enabling epitope-specific ADCs with enhanced therapeutic indices [59,150,151]. This paradigm shift places in silico methodologies as indispensable catalysts for next-generation ADC payload innovation.

The future of ADC development lies in the convergence of innovative payload engineering, precise linker design, and advanced antibody optimization—each increasingly driven by in silico methodologies. The integration of artificial intelligence, molecular dynamics, and structural modeling has enabled rational design workflows that were previously unattainable, accelerating the identification of stable, tumor-selective ADC candidates with improved therapeutic indices. Nevertheless, significant challenges remain, particularly in overcoming tumor heterogeneity, minimizing off-target toxicity, and ensuring efficient tissue penetration. Addressing these obstacles will require a multidisciplinary approach that leverages the chemical diversity of natural products, the predictive power of computational modeling, and the clinical insight gained from next-generation ADC trials. As the field continues to evolve, the synergy between natural compound discovery and AI-guided engineering holds promise for expanding the therapeutic reach of ADCs and transforming them into precision tools against a broader spectrum of cancers.

## Data Availability

Data is contained within the article material.

## Abbreviations

ABC	ATP-binding cassette
ADCC	Antibody-dependent cell-mediated cytotoxicity
ADC	Antibody-drug conjugate
ADMET	Absorption, Distribution, Metabolism, Excretion, and Toxicity
AML	Acute myeloid leukemia
AOC	Antibody oligonucleotide conjugate
cBu	Cyclobutane-1,1-dicarboxamide
CDR	Complementarity-determining region
CEA	Carcinoembryonic antigen
CH	Heavy chain constant domain
CL	Light chain constant domain
CPP-scFv	Cell-penetrating peptide single-chain variable fragment
DAR	Drug-Antibody ratio
DM1	Mertansine
DXd	Deruxtecan
ECM	Extracellular matrix
EGFR	Epidermal growth factor receptor
Fab	Antigen-binding fragment
Fc	Crystallizable fragment
FcRn	Neonatal Fc receptor
Fv	Variable fragment of antibody
GCC	Guanylyl cyclase C
GSH	Glutathione
HCs	Heavy chains
HER2	Human Epidermal Growth Factor Receptor 2
H3-OPT	CDR-H3 optimization toolkit
HR	Homologous recombination
IC50	Half-maximal inhibitory concentration
IgG	Immunoglobulin G
LC	Light chain
Lys-MCC-DM1	Lysine-linked maytansinoid metabolite
mAb	Monoclonal antibody
MDR1	Multidrug resistance protein 1
MD	Molecular dynamics
MMAE	Monomethyl auristatin E
MSA	Multiple Sequence Alignment
NAMPT	Nicotinamide phosphoribosyltransferase
NHEJ	Non-homologous end joining
PABC	Para-aminobenzyl carbamate
PBD	Pyrrolobenzodiazepine
PEG	Polyethylene glycol
pLDDT	Predicted Local Distance Difference Test
PROTAC	Proteolysis Targeting Chimera
RAbD	RosettaAntibodyDesign
scFv	Single-chain variable fragment
SMCC	Succinimidyl-4-(N-maleimidomethyl) cyclohexane-1-carboxylate
STING	Stimulator of Interferon Genes
TAP	Therapeutic Antibody Profiler
T-DM1	Trastuzumab emtansine
TME	Tumor microenvironment
VH	Variable heavy chain domain
VL	Variable light chain domain
